# Ultrasound-Guided Minimal Invasive Carpal Tunnel Release: An Optimized Algorithm

**DOI:** 10.1007/s00270-021-02789-2

**Published:** 2021-02-24

**Authors:** Alexander Loizides, Sarah Honold, Elisabeth Skalla-Oberherber, Leonhard Gruber, Wolfgang Löscher, Bernhard Moriggl, Marko Konschake, Hannes Gruber

**Affiliations:** 1 Department of Radiology, Medical University Innsbruck, Anichstrasse 35, 6020 Innsbruck, Austria; 2 Department of Neurology, Medical University Innsbruck, Anichstrasse 35, 6020 Innsbruck, Austria; 3 Institute of Clinical and Functional Anatomy, Medical University Innsbruck, Muellerstrasse 59, 6020 Innsbruck, Austria

**Keywords:** Carpal tunnel syndrome, Minimal invasive carpal tunnel release, Ultrasound-guidance

## Abstract

**Purpose:**

To present a safety-optimized ultrasound-guided minimal invasive carpal tunnel release (CTR) procedure.

**Materials and Methods:**

104 patients (67 female, 37 male; mean age 60.6 ± 14.3 years, 95% CI 57.9 to 63.4 years) with clinical and electrophysiological verified typical carpal tunnel syndrome were referred for a high-resolution ultrasound of the median nerve and were then consecutively assigned for an ultrasound-guided CTR after exclusion of possible secondary causes of carpal tunnel syndrome such as tumors, tendovaginitis, ganglia and possible contraindications (e.g., crossing collateral vessels, nerve variations). Applying a newly adapted and optimized algorithm, basing on the work proposed by Petrover et al. CTR was performed using a button tip cannula which has several safety advantages: On the one hand, the button tip cannula acts as a blunt and atraumatic guiding splint for the subsequent insertion of the hook-knife, and on the other hands, it serves as a “hydro-inflation”-tool, i.e., a fluid-based expansion of the working-space is warranted during the whole procedure whenever needed.

**Results:**

In all patients, successful releases were confirmed by the depiction of a completely transected transverse carpal ligament during and in the postoperative ultrasound-controls two weeks after intervention. All patients reported markedly reduction of symptoms promptly after this safety-optimized ultrasound-guided minimal invasive CTR and at the follow-up examination. No complications were evident.

**Conclusion:**

The here proposed optimized algorithm assures a reliable and safe ultrasound-guided CTR and thus should be taken into account for this minimal invasive interventional procedure.

**Supplementary Information:**

The online version contains supplementary material available at (10.1007/s00270-021-02789-2).

## Introduction

Carpal tunnel syndrome (CTS) is the most frequent compressive neuropathy, caused by compression of the median nerve at the level of the transverse carpal ligament (TCL) [1–3]. Diagnosis of CTS is mainly based on typical clinical symptoms, electrodiagnostic testing and high-resolution ultrasound (HRUS) [4].

In mild forms of CTS conservative treatment such as splint wearing at night, physiotherapy or steroid injections can initially be considered, whereas in severe forms or in patients with failed non-surgical treatment, carpal tunnel release (CTR) is indicated with patients showing an improvement in more than 90% [5, 6] of the cases.

Open CTR has been performed successfully for many years [7] with a trend, however, toward less-invasive procedures [7–11]. Over the recent years – most notably by Petrover et al. [8] – the safety and efficacy of real-time HRUS-guided minimally invasive CTR procedures have been demonstrated, leading to a lower invasiveness, less tissue trauma and a lower rate of procedural complications [8, 9, 12–15]. We hereby present a modified HRUS-guided minimally invasive CTR procedure utilizing as a novelty a blunt button tip cannula, which shows several advantages regarding procedural performance and median nerve safety.

## Material and Methods

### Demographics

One-hundred and four patients (67 female, 37 male; mean age 60.6 ± 14.3 years, 95% CI 57.9 to 63.4 years) were included. No patients had to be excluded due to anatomical variations (Table 1).

**Table 1:** Demographic data and CTS findings

	Results	95% CI^$^
Age	60.6 ± 14.3 years	57.9–63.4 years
Sex: male/female	37/67 (37.6/64.4%)	–
Affected side: right	60 (57.7%)	–
CTS severity		
“mild”	10 (12.1%)	–
“moderate”	43 (51.8%)	–
“severe”	30 (36.1%)	–
Median nerve CSA	20.2 ± 5.4 mm^2^	19.0–21.3 mm^2^
Wrist-to-forearm ratio	2.1 ± 0.7	2.0–2.3

^$^Confidence interval

### Carpal tunnel release procedures

All consecutive patients referred to our department for HRUS-guided CTR with clinically and electrophysiologically verified CTS and failed conservative treatment from December 2019 to October 2020 were included in this study. All procedures performed were in accordance with the ethical standards of the institutional and/or national research committee and with the 1964 Helsinki declaration and its later amendments or comparable ethical standards.

Prior to the intervention all patients underwent HRUS-assessment to exclude possible anatomic contraindications (e.g., crossing collateral vessels, tumors) or nerve variations, such as Berrettini branches (anastomoses between the common digital nerves of the median and ulnar nerve) or Riche-Cannieu anastomoses (anastomoses between the thenar motor branch of the median nerve and the deep branch of the ulnar nerve) [16]. Median nerve cross-section areas (CSA) were measured to calculate the wrist-to-forearm ratio [17]. For the according written consent, a weighed explanation of the planned procedure including potential advantages and risks as well as the novel nature of the procedure was addressed.

#### Preoperative preparations

All patients were placed in a supine position with the respective arm extended at 90 degrees and the hand positioned supinated on a supporting cushion. Two belts were used to loosely fix the forearm and the extended fingers, respectively. Under strict aseptic conditions (with the intervening specialist wearing a mask, sterile gloves and a sterile gown) the patient’s arm was cleansed, and sterile drapes were used to cover the region. Using a Canon Aplio i800® system (Canon Medical Systems, Tokyo, Japan) with a linear 18L7 transducer (including a sterile transducer cover and sterile gel) the median nerve, ulnar artery and the superficial palmar arch were marked with a sterile marker on the skin defining thereby the safe release path— “safe zone”(15).

#### Interventional technique

**Fig. 1:**
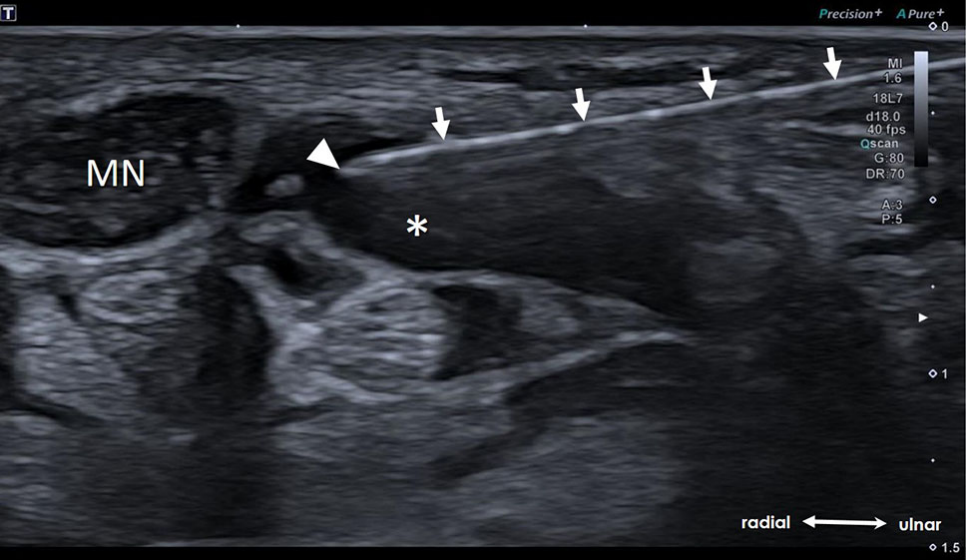
US-axial scan depicting the hydrodissection of the median nerve (MN) using a 21G needle (arrows) and the needle tip (arrowhead) within the “expanded” perineural space (asterisk)

**Fig. 2:**
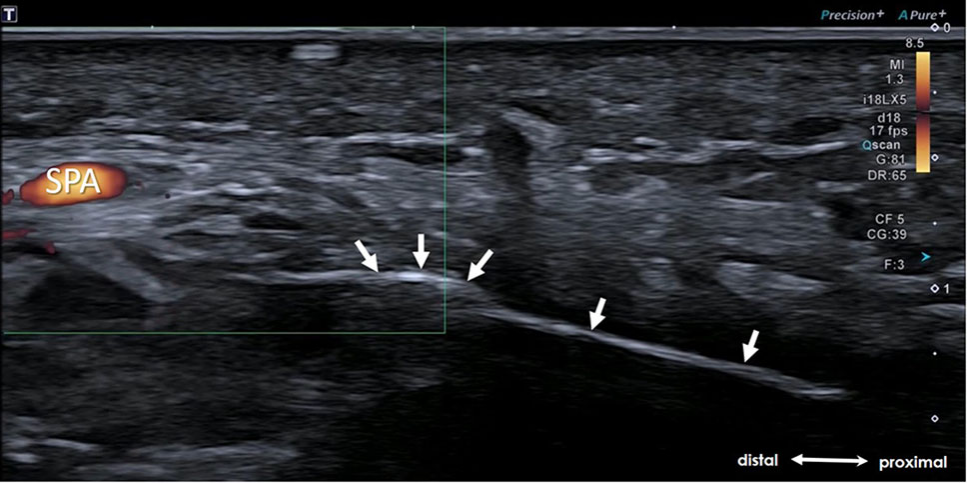
Longitudinal US-scan illustrating the positioning of the button tip cannula (arrows) at the distal reach of the safe zone. Superficial palmar arch (SPA)

**Fig. 3:**
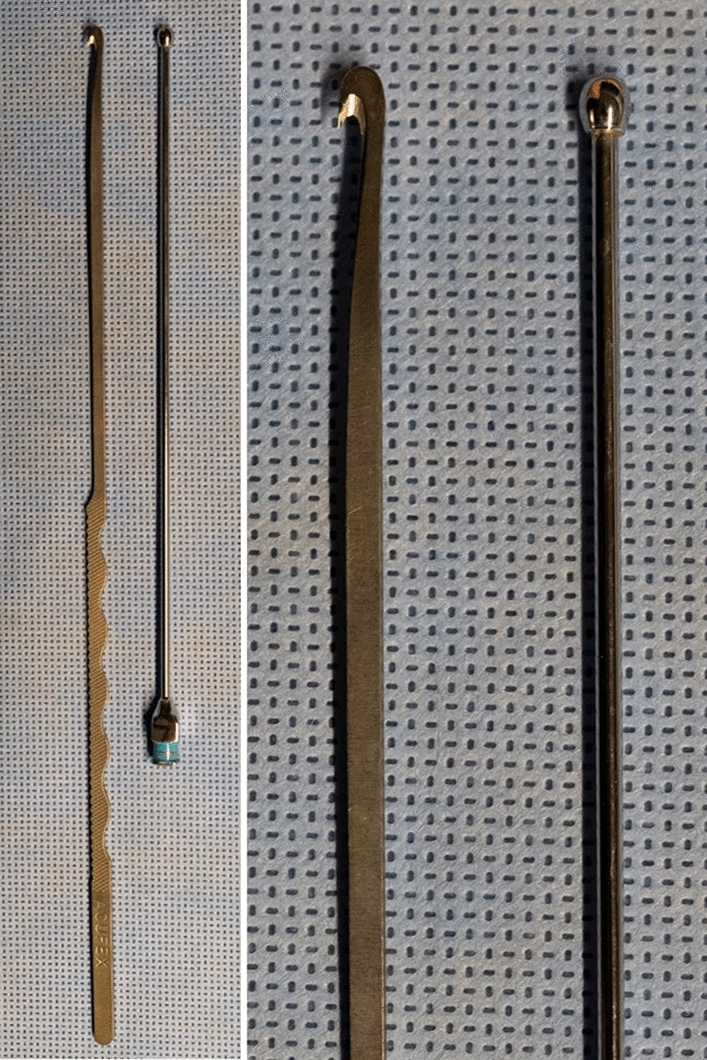
Depiction of the hook-knife (Acufex® 3.0-mm hook-knife, Smith & Nephew PLC, London, England) (left) and the button tip cannula (Keysurgical®, Lensahn, Germany) (right)

**Fig. 4:**
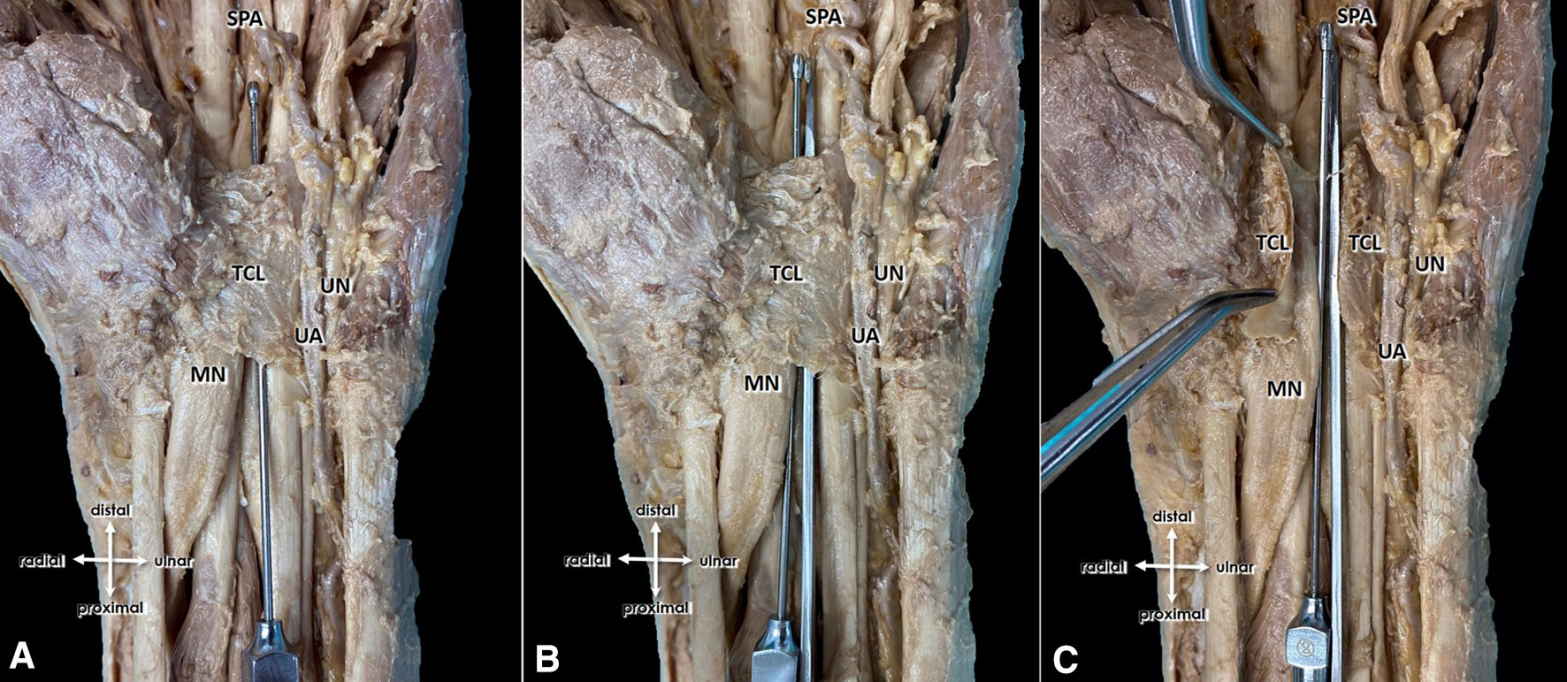
**A** Anatomic specimen depicting the button tip cannula within the carpal tunnel. Median nerve (MN), ulnar nerve (UN), ulnar artery (UA), transverse carpal ligament (TCL), superficial palmar arch (SPA). **B** After insertion of the hook-knife ulnar to the button tip. **C** Position of the instruments within the carpal tunnel with a transected TCL. Note that the MN in this specimen shows marked enlargement typical in patients with CTS

**Fig. 5:**
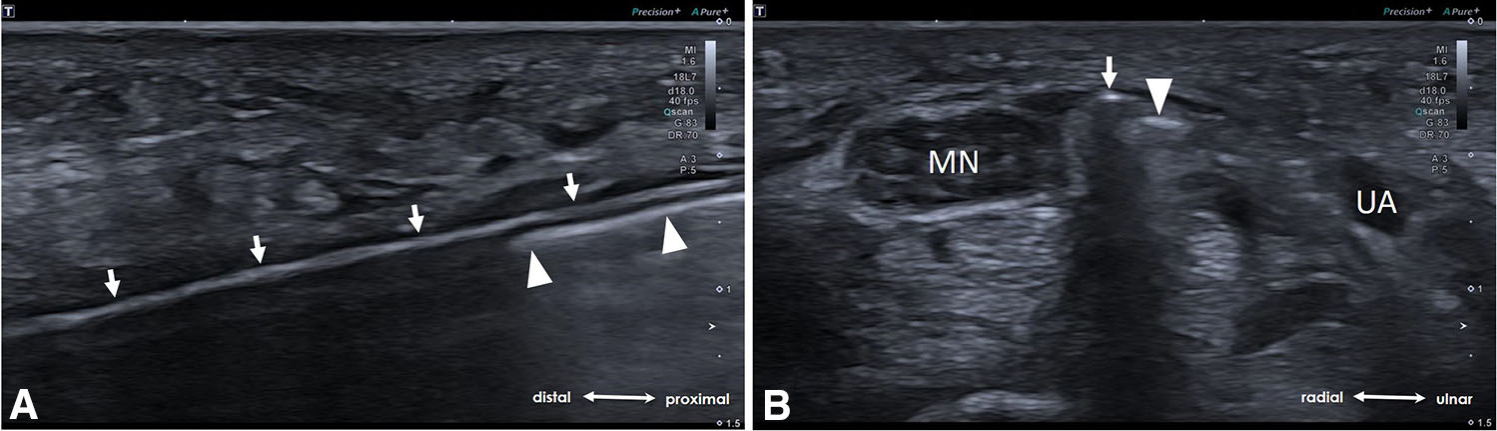
**A** Longitudinal US-scan demonstrating the button tip cannula within (arrows) and the hook-knife (arrowheads) entering the carpal tunnel. **B** Axial US-scan depicting the “Double-Dot Sign” formed by cross-section of the button tip cannula (arrow) and the hook-knife (arrowhead). Median nerve (MN), ulnar artery (UA)

**Fig. 6:**
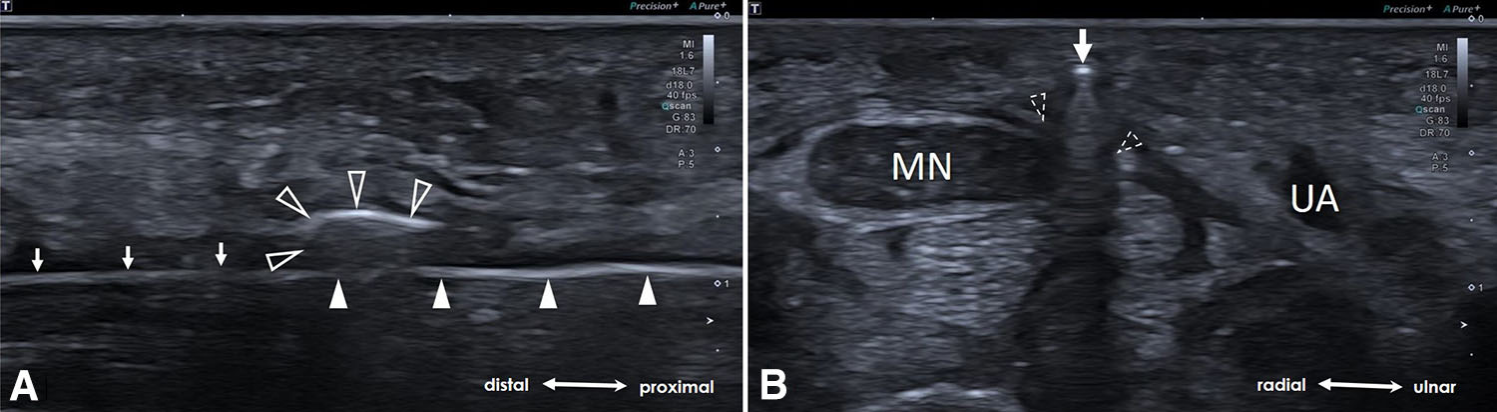
**A** Longitudinal US-scan illustrating the cutting of the TCL by retraction of the hook-knife (arrowheads). Hook (void arrowheads). The button tip cannula (arrows) which serves as a guiding tract, remains within the carpal tunnel during the whole release. **B** Axial US-scan depicting a successful release by “palpation” of the button tip cannula (arrow) through the transected TCL (dotted arrowheads). Median nerve (MN), ulnar artery (UA)

1. Subcutaneous local anesthesia at the incision site (approx. 1.5 cm proximal to the dominant flexor crease of the ulnar sided wrist) with a 25G needle and 2 ml of 0.5% Carbostesin (Bucain®).
2. Hydrodissection of the median nerve with 5 ml of 0.5% Carbostesin (Bucain®) after insertion of a 21G needle into the perineural space from the ulnar aspect while scanning under real-time transaxial guidance.(Fig. 1).
3. Small incision (approximately 3 mm) of the skin and subcutaneous fascia at the intended entry point of the hook-knife (approx. 1.5 cm proximal to the dominant flexor crease of the ulnar sided wrist) by a sterile standard 11 mm disposable scalpel.
4. Advancement of a disposable button tip 16F metal cannula (Keysurgical®, Lensahn, Germany) longitudinally under HRUS-control through the safe zone up to the distal edge of the TCL (superficial palmar arch visible in this scan plane) (Fig. 2). The blunt button tip cannula serves as a guiding track and is swayed within the safe zone, to free the carpal tunnel (CT) from possible adhesions and guide the subsequent insertion of the knife.
5. Further injections of sterile physiologic saline through the button tip cannula to additionally widen the perineural safe zone just before inserting the hook-knife whenever necessary guaranteeing a sufficient widening of the safe zone during the whole dissection procedure.
6. Subsequent advancement of a hook-knife (Acufex® 3.0-mm hook-knife, Smith & Nephew PLC, London, England) parallel and ulnar to the button tip cannula with the cutting hook oriented ulnar, until the knife-tip reaches the level of the tip of the cannula (“dead end” of the safe procedure) (Figs. 3, 4), depicting a “Double-Dot Sign” in an axial scan plane within the safe zone deep to the TCL (Fig. 5).
7. The knife is then rotated under HRUS control and “hooks” the distal edge of the TCL – a transection of the TCL is subsequently achieved by continuously retracting the knife longitudinally under HRUS-vision(Fig. 6A).
8. Removal of the hook-knife after verification of the successful release by tilting the button tip cannula through the transected TLC (Fig. 6B).
9. Covering of the skin-incision with a band-aid (Steri-Strip®, 3 M Deutschland GmbH Health Care Business) and a compression bandage.

After the procedure, patients were advised to use their treated hand normally and to perform specific exercises, which were given to them immediately after the procedure.

## Results

In all patients (*n* = 104, 100.0%), a technically successful CT-release could be documented on the basis of (a) a clearly HRUS-proven transected TLC and (b) the movement of the button tip cannula through the gap of the transected TCL. In all patients, 5 ml of physiologic saline was injected through the button tip cannula to widen initially the perineural safe zone. In 16 patients, additional 5 ml of saline was used to widen further a “narrow” perineural space just before inserting the hook-knife. In the postoperative HRUS controls two weeks after intervention (always done by an experienced nerve sonographer not engaged in the HRUS-guided procedure), a fully transected TCL was clearly defined and documented in each patient. Other than sparse hematoma at the incision site, no major complications (vascular or nerve injury) were evident. Applying a simplified Boston Carpal Tunnel Syndrome Questionnaire (BCTQ) all patients reported a marked reduction of their specific symptoms promptly after CT-release and at the follow-up examination.

## Discussion

Various studies have been published describing techniques for median nerve decompression by TCL release (5–15). In our study, a successful release was also demonstrated in all of our patients. Although not formally studied, an immediate reduction of their specific symptoms was additionally observed promptly after CT-release and during the follow-up examination. This positive effect with quick improvement of hand function and reduction of hand discomfort was also demonstrated in a recent study by Kamel et al. [18].

The technique described by Petrover et al. [8] was optimized in our study by initially inserting a button tip cannula prior to the hook-knife which offers clear advantages: On the one hand, a blunt cannula is used to widen the safe zone of the CT and to free it from possible adhesions and serves on the other hands as a guiding splint for the subsequent insertion of the hook-knife which enables a relative smooth insertion of the knife along the button tip cannula: this is especially in elderly patients with, e.g., arthritic changes and habitual radiocarpal extension a great advantage, as in such cases the hook-knife alone often deviates or slides below the median nerve with a greater risk of unintended nerve injury by repositioning. Additionally the button tip cannula serves as a clear marker indicating the most distal reach of the safe zone prior to the insertion of the knife avoiding thus any unintended injury of the superficial palmar arch artery when advancing the hook-knife beyond that point. A further advantage of the button tip cannula is the possibility of “hydro-inflating” the safe zone at any time during our CTR procedure if needed. Initially injected local anesthetics, displace within the CT: Due to the confined space and proximal and distal open nature of the CT, the injected fluid spreads and thus tunnel expansion decays gradually and rather prompt during the procedure, which might complicate the insertion of the hook-knife. This problem is minimized as additional saline can be injected at any time especially immediately before starting the cutting procedure itself. At the end of the procedure, documentation of a successfully transected TCL is mandatory and can be done by either moving the hook-knife through the transected TCL or as described by Petrover et al. [8] by secondarily injecting physiologic saline to distend and thus proof the released TCL or by introducing a cannula into the tunnel by a separate step to directly show the release by “HRUS-palpation”: Although the convex side of the hook-knife is blunt, the tip of the hook is spiky and may again unintendedly injure surrounding tissues by “palpating” the divided TCL. Thus, in our proposed algorithm, the explicit “procedure-success-control” can simply be done by using the atraumatic button tip cannula being still in place avoiding any unnecessary trauma.

Although the intention of our study was to describe this new modified algorithm which definitely optimizes the technique proposed by Petrover regarding procedural performance and safety, a limiting factor was the missing inclusion of a control group.

## Conclusion

This proposed algorithm which is an evolution of a previously described technique by Petrover et al. [8] assures a reliable and safe ultrasound-guided CTR and thus should be considered when performing percutaneous HRUS-guided carpal tunnel release.

## Supplementary Information

Supplementary file1 (mp4 50359 kb)
